# Anacardic acid mitigates liver fat accumulation and impaired glucose tolerance in mice fed a high‐fat and high‐sucrose diet

**DOI:** 10.1002/fsn3.1322

**Published:** 2020-01-17

**Authors:** Sangwon Chung, Eun Ju Shin, Hyo‐Kyoung Choi, Jae Ho Park, Jin‐Taek Hwang

**Affiliations:** ^1^ Korea Food Research Institute Wanju‐gun Jeollabuk‐do Korea; ^2^ Department of Food Biotechnology University of Science & Technology Daejeon Korea

**Keywords:** anacardic acid, antidiabetes, high‐fat and high‐sucrose diet, homeostasis model assessment of insulin resistance, mice

## Abstract

In this study, we evaluated the effects of anacardic acid (AA), a phenolic lipid found in cashew nuts (*Anacardium occidentale*), on metabolic disorders related to obesity, fatty liver disease, and diabetes using both in vitro and in vivo models. The application of AA led to a reduction in lipid accumulation in 3T3‐L1 cells without observable cytotoxicity. Results from Western blot analysis revealed that AA treatment also led to decreased expression of fatty acid synthase and peroxisome proliferator‐activated receptor gamma. In vivo studies were performed to evaluate the effects of AA treatment on fatty liver disease and diabetes. Mice fed a high‐fat and high‐sucrose diet had significantly higher body and liver weights, and higher levels of liver fat, cholesterol, fasting glucose, and homeostasis model assessment of insulin resistance (HOMA‐IR). However, 12 weeks of oral treatment with 500 μg/kg BW AA slowed down lipid accumulation rates in the liver and mitigated insulin resistance in these mice. Thus, AA may reduce lipid levels and have an antidiabetic effect.

## Graphical abstract

Anacardic acid inhibits lipid accumulation in 3T3‐L1 cells. Anacardic acid reduces lipid accumulation in mice fed high‐fat and high‐sucrose diet. Anacardic acid mitigates insulin resistance in mice fed high‐fat and high‐sucrose diet.

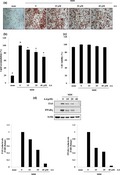

## 1 INTRODUCTION

Metabolic dysfunctions such as lipogenesis and insulin resistance are major contributors to obesity, type 2 diabetes, and cardiovascular diseases, which are major global health concerns (Kahn, Hull, & Utzschneider, 2006; Milic, Lulic, & Stimac, 2014). Nonalcoholic fatty liver disease (NAFLD) is also considered a metabolic syndrome as it shares common mechanisms with pathologies such as insulin resistance (Anstee, Targher, & Day, 2013). NAFLD is mainly caused by excessive free fatty acids and chronic inflammation in the adipose tissue (Milic et al., 2014). As fat accumulates in the liver, insulin signaling declines, leading to abnormal hepatic metabolism (Sookoian & Pirola, 2017).

Several agonists play important roles in the treatment of metabolic diseases. For example, peroxisome proliferator‐activated receptors (PPARs) and fatty acid synthase (FAS) agonists have been shown to regulate lipid and glucose metabolism, as well as inflammation (Bensinger & Tontonoz, 2008; Botta et al., 2018; Gross, Pawlak, Lefebvre, & Staels, 2017; Moseti, Regassa, & Kim, 2016). However, agonists come with several side effects such as weight gain, cardiovascular risk, and hepatopathy. In contrast, medicinal plants have long been used for medical treatments and are considered a safe alternative. Thus, medicinal plants may be useful for preventing metabolic disorders.

Natural ingredients such as herbal extracts may alleviate metabolic diseases by modulating target molecules such as PPAR‐γ and FAS. For example, the ethanol extracts of the peels of *Citrus junos* Tanaka and *Prunus mume* contain abundant flavonoids, which activate PPAR‐γ to stimulate glucose uptake in C2C12 cells (Kim et al., 2013; Shin et al., 2013). In addition, epigallocatechin‐3‐gallate (EGCG) has been shown to attenuate adipogenesis, thus downregulating PPAR‐γ and FAS expression in 3T3‐L1 cell lines (Wu et al., 2017).

Anacardic acid (AA), which is isolated from the cashew plant (*Anacardium occidentale*), also alleviates metabolic dysfunction. Cashew nut extracts and AA have been shown to increase glucose uptake by upregulating plasma membrane glucose transporters in vitro (Tedong et al., 2010). However, AA effects have not been well studied, particularly in in vivo models. Therefore, in this study, we investigated whether AA affects lipid accumulation in 3T3‐L1 cells and whether AA treatment mitigates fatty liver disease and insulin resistance in mice. Changes in liver fat and glucose levels, as well as lipid and insulin parameters, were measured in mice fed a high‐fat and high‐sucrose diet.

## 2 MATERIALS AND METHODS

### 2.1 Materials

We purchased 3T3‐L1 cells from the American Type Culture Collection. Dulbecco's modified Eagle's medium (DMEM) and fetal bovine serum (FBS) were purchased from WelGENE. Anacardic acid, insulin, 3‐isobutyl‐1‐methylxanthine (IBMX), and dexamethasone were purchased from Sigma‐Aldrich. Anti‐FAS and PPAR‐γ antibodies were purchased from Cell Signaling Technology, and anti‐β‐actin was purchased from Bethyl Laboratories.

### 2.2 Adipocyte differentiation and Oil Red O staining

The 3T3‐L1 cells were cultured in DMEM supplemented with 10% (v/v) calf serum, antibiotics, and antimycotics. Cells grown at 100% in a 6‐well plate were cultured for an additional 2 days and then transferred to a medium supplemented with 0.5 mM IBMX, 10 μg/ml insulin, 0.5 μM dexamethasone, and heat‐inactivated 10% FBS (MDI medium). Cells were cultured in the MDI medium for 2 days to induce cell differentiation. Subsequently, cells were cultured in 10 μg/ml insulin in the presence or absence of AA. This medium was replaced every 2 days. For Oil Red O staining, cells were first washed twice with phosphate‐buffered saline (PBS) and then fixed in 2 ml 3.7% paraformaldehyde solution (diluted with PBS) for 5 min at room temperature. The solution was then removed, and the cells were fixed again for 1 hr, after which the paraformaldehyde solution was removed, and the cells were dried at room temperature. The dried cells were stained in Oil Red O working solution for 10 min and washed five times with distilled water. Stained cells were photographed under a microscope. Then, 100% isopropanol was added to elute the accumulated lipids in the cells, and the absorbance of the subsequent liquid was measured at 510 nm using a Micro plate reader (Molecular Devices).

### 2.3 Cell viability test

Cell viability was measured using 3‐(4,5‐dimethylthiazol‐2‐yl)‐2, 5‐diphenyltetrazolium bromide (MTT) solution. After the lipids were eluted from the cells, 100 μg/ml MTT solution was added to the cells and they were incubated for 3 hr, forming a blue product. The solution was then discarded, and the precipitate was solubilized using dimethyl sulfoxide (DMSO). The absorbance of the resulting solution was measured at 540 nm using a Micro plate reader (Molecular Devices).

### 2.4 Western blot analysis

Proteins were extracted using a radioimmunoprecipitation assay buffer that contained protease and phosphatase inhibitors. Protein quantification was carried out using the Bradford reagent (Biosesang). Electrophoresis was performed using sodium dodecyl sulfate–polyacrylamide gel with 30 μg of proteins, and samples were then transferred to nitrocellulose membranes. Western blot analysis and band detection were performed using an EZ‐Western chemiluminescence detection kit (Dongin Biotech) according to the manufacturer's instructions.

### 2.5 Animal experiments and diet‐induced obesity

Animal experiments were performed at INVIVO Inc.. We obtained 4‐week‐old male C57BL/6 mice (Samtakobio) and housed them in a climate‐controlled environment at 22°C and relative humidity of 50% under a 12‐hr light/dark cycle for 1 week. The mice were then randomly divided into four groups of 10 mice each. Mice were fed (a) a normal diet (ND); (b) a high‐fat and high‐sucrose (HFS) diet; (c) a HFS diet with 250 μg/kg BW AA; and (d) a HFS diet with 500 μg/kg BW AA. Diets were purchased from Research Diets; we used diet D12450B (10% of total calories from fat) as the ND and diet D12079B as the HFS diet. The composition of the HFS diet is described in Table 1. The AA was dissolved in DMSO and diluted with corn oil (C8276; Sigma‐Aldrich). Mice were fed these diets for 12 weeks while having free access to autoclaved tap water. Food intake was quantified once a week by measuring the amount of food left the day after feeding. At the end of the experiment, the mice were sacrificed, and their blood was sampled from the abdominal vein after ether anesthesia. Liver and epididymal fat was also extracted and weighed. One liver lobe from each mouse and the epididymal fat were fixed in 4% formalin for further analysis. Our protocol for this animal experiment was approved (WKU17‐101) by the Institutional Care and Use Committee at Wonkwang University, Iksan, Republic of Korea.

**Table 1:** Composition of the high‐fat and high‐sucrose (HFS) diet

Ingredient	Weight (g)	Caloric value (Kcal)
Casein, 80 mesh	195	780
DL‐methionine	3	12
Corn starch	50	200
Maltodextrin 10	100	400
Sucrose	341	1,364
Cellulose	50	0
Milk fat, anhydrous	200	1,800
Corn oil	10	90
Mineral Mix S10001	35	0
Calcium carbonate	4	0
Vitamin Mix V10001	10	40
Choline bitartrate	2	0
Cholesterol*	1.5	0
Ethoxyquin	0.04	0
Total	1,001.54	4,686

* United States Pharmacopeia reference standard.

### 2.6 Glucose tolerance test (GTT)

Mice were starved for more than 8 hr, and their fasting blood glucose levels were measured by inserting a blood glucose meter (Autochek DIATech) into a vein. Blood glucose levels were measured again after 30 min, and 2 g/kg BW glucose was added to the results of all mice, as recommended in the experimental protocol. Subsequently, blood glucose levels were measured through the subcutaneous vein every 30 min for 120 min. From these results, the area under the curve (AUC) was calculated, and the blood sugar change was analyzed for each treatment group.

### 2.7 Hematoxylin and eosin staining

Liver tissues were excised and fixed in 10% formalin solution. Tissues were then embedded in paraffin, cut into sections of 5‐μm thickness, and stained with Hematoxylin and eosin (H&E). The prepared tissues were examined using an optical microscope.

### 2.8 Measurement of biochemical parameters

Sampled blood was coagulated at room temperature for 30 min and then centrifuged at 3,000 rpm for 10 min to separate the blood serum. The serum was analyzed by Green Cross Medical Foundation (GC Labs) for total cholesterol (TC), triglyceride (TG), low‐density lipoprotein cholesterol (LDL‐C), and high‐density lipoprotein cholesterol (HDL‐C) content. Serum insulin content was analyzed using an ALPCO ELISA kit.

### 2.9 Statistical analyses

All results from the animal experiments are presented as the mean ± standard error (SE). Results were compared using one‐way analysis of variance (ANOVA), followed by Duncan's test using SPSS version 12.0 (SPSS Inc.). Differences were considered statistically significant at *p* < .05.

## 3 RESULTS

### 3.1 Effect of AA on adipocyte differentiation

**Figure 1:**
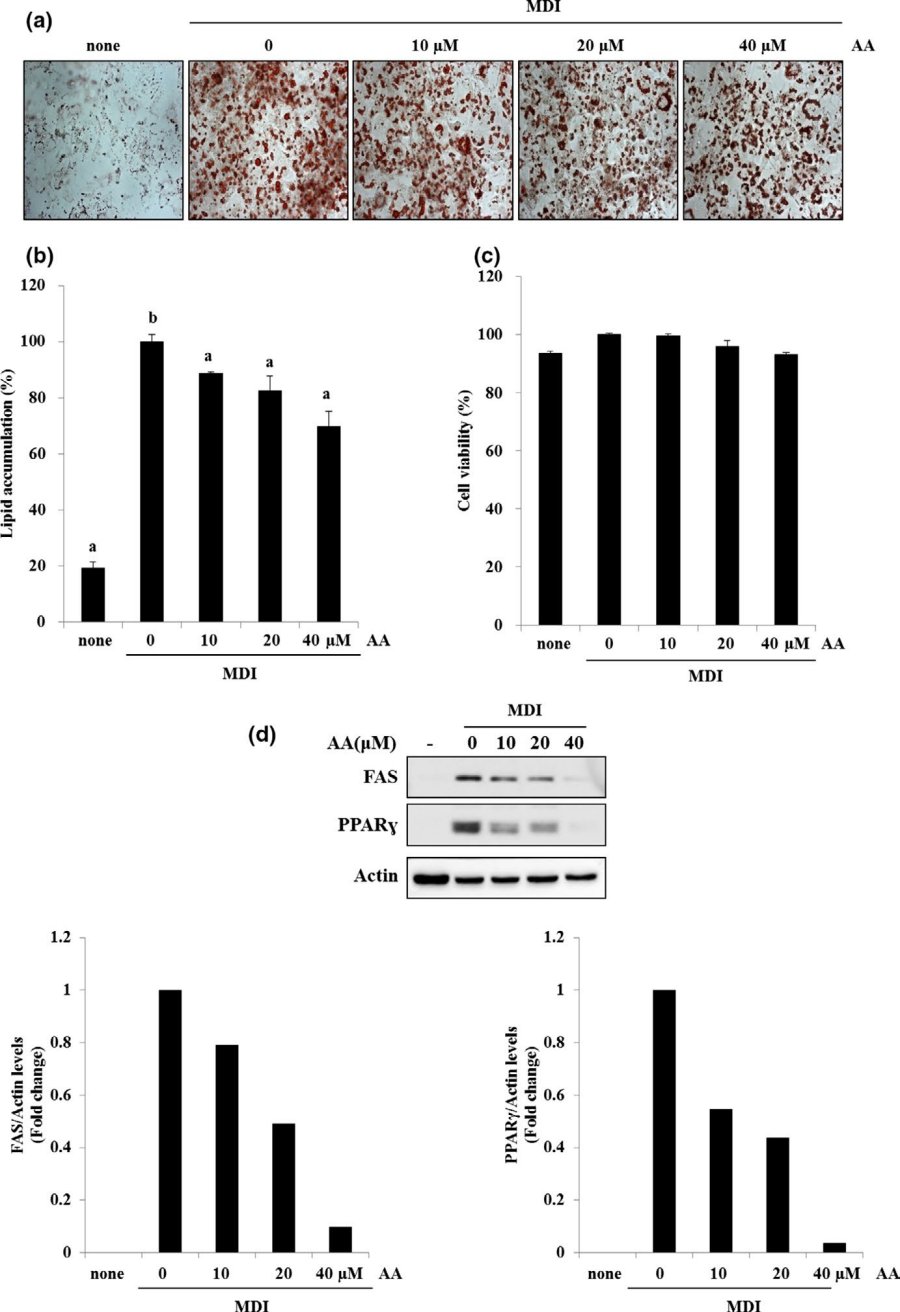
Effects of anacardic acid (AA) on adipocyte differentiation and expression of lipid‐related proteins. (a, b) Cells from the 3T3‐L1 line were first treated with a differentiation medium (MDI). AA was then added to half the samples at various doses. All cells were stained with Oil Red O solution. (c) No cytotoxicity was observed for 3T3‐L1 cells treated with AA up to a concentration of 40 μM. (d) Western blot results. AA attenuated the expression levels of fatty acid synthase (FAS) and peroxisome proliferator‐activated receptor‐γ (PPAR‐γ), which were initially upregulated by MDI treatment (the different lowercase letters indicate significant differences at 0.05 level; *p* < .05)

We used the adipocyte differentiation model to evaluate the effect of AA treatment on lipid accumulation. From the examination of Oil Red O‐stained cells, AA treatment significantly reduced lipid accumulation stimulated by the MDI medium in a dose‐dependent manner (Figure 1a,b). Reductions were observed at AA concentrations of 10, 20, and 40 μM. No cytotoxicity was observed in the 3T3‐L1 cells treated with AA (Figure 1c). Lipid accumulation was accompanied by the upregulation of adipogenesis‐related markers such as PPAR‐γ and FAS. PPAR‐γ and FAS expression increased in adipocytes that had differentiated after MDI induction, but decreased in cells treated with AA in a dose‐dependent manner (Figure 1d). Our results reveal that AA is effective at inhibiting lipid accumulation by reducing the expression of lipid‐related proteins such as PPAR‐γ and FAS.

### 3.2 Effects of AA on body and liver weights, food intake, and liver and epididymal fat in mice fed an HFS diet

**Figure 2:**
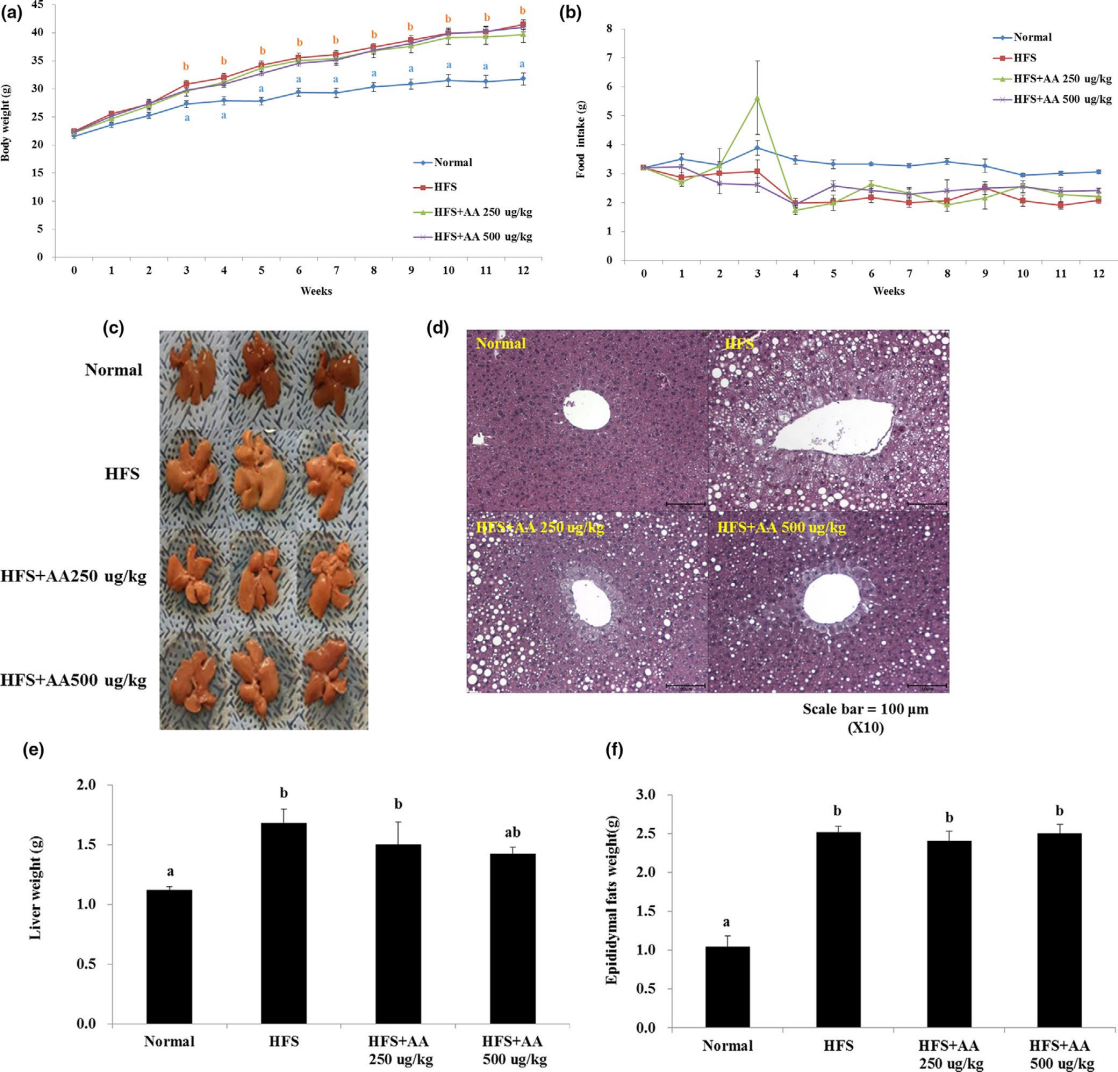
Effects of AA on body weight, food intake, and liver and epididymal fat in mice fed a high‐fat and high‐sucrose (HFS) diet. (a) Mice fed a HFS diet had significantly higher body weight than mice fed a normal diet (ND). However, the consumption of 500 μg/kg BW AA slightly reduced the body weights of HFS‐treated mice. (b) Food intake (g) was significantly lower in HFS‐treated mice than in ND mice. However, no significant differences were observed between mice on a HFS diet and mice that consumed both a HFS diet and AA. (c) Upon visual observation, HFS‐treated mice accumulated significantly more liver fat than ND mice; however, this additional fat was reduced when AA was consumed. (d) Examination of tissues stained with hematoxylin and eosin indicated that the HFS diet led to an increase in the accumulation of liver fat, which was subsequently reduced when AA was consumed. (e) Liver weights were slightly reduced in HFS‐treated mice that also consumed AA. (f) Epididymal fat weights were significantly higher in HFS‐treated mice. AA consumption did not affect epididymal fat content (the different lowercase letters indicate significant differences at 0.05 level; *p* < .05, *n* = 7, data are presented as the mean ± standard error (SE))

We investigated whether AA treatment can lower lipid levels and mitigate obesity‐related diseases in vivo using mice fed a HFS diet. Mice fed the HFS diet had significantly higher body and liver weights, and higher levels of liver and epididymal fat, than ND mice. AA treatment led to slightly reduced liver weights and significantly reduced levels of liver fat in HFS‐treated mice. However, AA had no effects on the body weights and epididymal fat levels of HFS‐treated mice (Figure 2a–f). Similar to results observed in the cell culture experiment, AA treatment reduced the liver weights and fat accumulation rates in HFS‐treated mice. We did not observe any differences in food intake among treatment groups (Figure 2b).

### 3.3 Effects of AA on the biochemical parameters of mice fed a HFS diet

**Figure 3:**
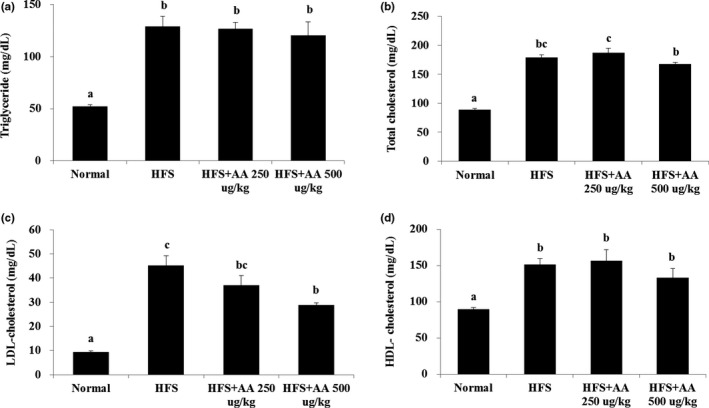
Effects of AA on levels of serum triglycerides (TGs), total cholesterol (TC), and low‐density lipoprotein (LDL‐C) and high‐density lipoprotein (HDL‐C) cholesterol in mice fed a HFS diet. (a) HFS‐treated mice had significantly higher TG levels than ND mice. Consumption of 500 μg/kg BW AA slightly reduced TG levels in HFS‐treated mice. (b) TC levels were significantly higher in HFS‐treated mice than in ND mice, but decreased slightly in the former group when AA was consumed. Levels of (c) LDL‐C and (d) HDL‐C were significantly higher in HFS‐treated mice than in ND mice, but LDL‐C significantly decreased in the group when 500 μg/kg BW of AA was consumed (the different lowercase letters indicate significant differences at 0.05 level; *p* < .05, *n* = 7, data are presented as the mean ± SE)

Serum TG, TC, LDL‐C, and HDL‐C levels were significantly higher in mice fed a HFS diet than in ND mice. AA treatment led to significant reductions in LDL‐C levels and slight reductions in TC levels in HFS‐treated mice. However, AA treatment did not significantly affect TG and HDL‐C levels in HFS‐treated mice (Figure 3a–d). Our results suggest that AA can reduce fat accumulation in the liver and LDL‐C levels induced by a HFS diet.

### 3.4 Effects of AA on fasting glucose levels and impaired glucose tolerance

**Figure 4:**
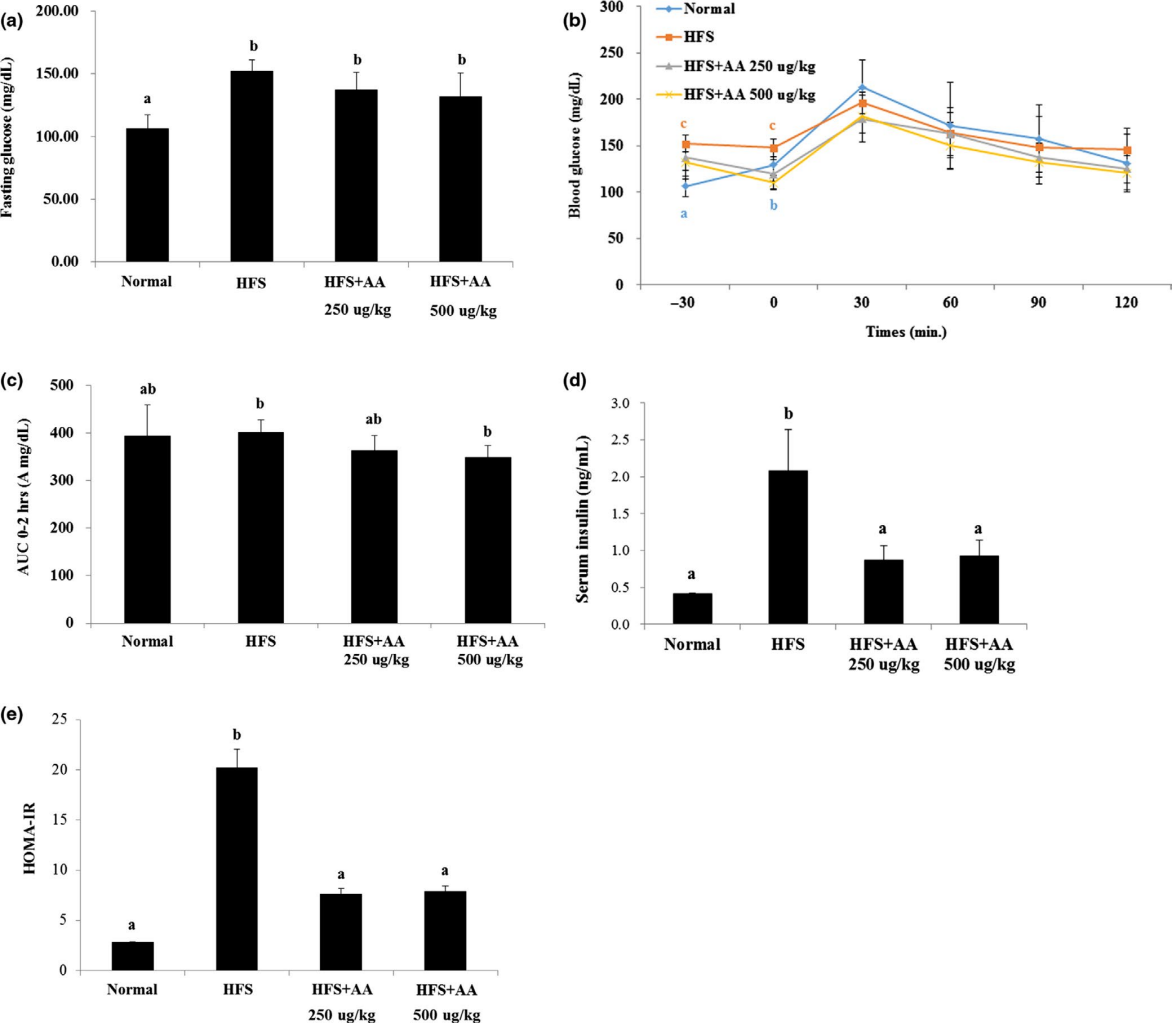
Effects of AA on fasting glucose levels and glucose intolerance in mice fed an HFS diet. (a) Fasting glucose levels of HFS‐treated and ND mice after 12 hr of starvation. (b) Results of glucose tolerance tests performed during the final weeks of the study. Details of the test are found in the Materials and Methods section. (c) Area under the curve (AUC) for each treatment. Mice fed a HFS diet had significantly higher levels of (d) serum insulin and (e) HOMA‐IR compared to ND mice, both of which were significantly reduced when AA was consumed (the different lowercase letters indicate significant differences at 0.05 level; *p* < .05, *n* = 7, data are presented as the mean ± SE)

We conducted GTT on mice that had fasted overnight. Fasting blood glucose levels were significantly higher in mice fed a HFS diet than in ND mice (Figure 4a). AA treatment slightly reduced fasting glucose levels in HFS‐treated mice (Figure 4a). In the GTT, no significant differences were observed among the treatment groups (Figure 4b), but the AUC was slightly lower for HFS‐treated mice exposed to AA than for mice fed the HFS diet alone (Figure 4c). In addition, serum insulin levels were significantly higher in HFS‐treated mice than in ND mice (Figure 4d), but AA treatment led to decreases in insulin levels in the former group. Finally, we conducted the homeostatic model assessment of insulin resistance (HOMA‐IR) using the results obtained in these experiments. HOMA‐IR levels were significantly higher in HFS‐treated mice than in ND mice (Figure 4e), but AA treatment led to a decrease in insulin resistance in the former group. These results indicate that AA can mitigate insulin resistance induced by HFS diet.

## 4 DISCUSSION

We first evaluated AA effects on lipid accumulation in vitro using 3T3‐L1 cells and then confirmed our results in vivo by examining the livers and glucose tolerance of mice fed a HFS diet. As seen in our experiments, AA inhibited adipocyte differentiation stimulated by MDI treatment in a dose‐dependent manner without inducing cytotoxicity in 3T3‐L1 cells. The inhibition stems from the reduced expression of several adipogenesis‐related markers such as PPAR‐γ and FAS. AA treatment also decreased the liver weights and liver fat, TC, and LDL‐C levels of mice fed a HFS diet. Additionally, AA treatment slightly decreased the fasting blood glucose levels of HFS‐treated mice and significantly decreased their serum insulin and HOMA‐IR levels. Overall, AA treatment reduced lipid accumulation in the liver and improved insulin resistance.

AA has been shown to prevent metabolic disorders in in vitro studies. For example, cashew seed extracts and AA induced adenosine monophosphate‐activated protein kinase and stimulated glucose uptake by upregulating plasma membrane glucose transporters in C2C12 cells (Tedong et al., 2010). However, AA did not induce the production of protein kinase B and insulin receptor phosphorylation (Tedong et al., 2010). In breast cancer cells, AA inhibited cell proliferation by modulating mitochondrial bioenergetics (Radde, Alizadeh‐Rad, Price, Schultz, & Klinge, 2016). Similar anticancer effects have also been observed in prostate cancer cells (Park et al., 2018; Tan et al., 2017). Therefore, AA, a natural compound, may be able to prevent metabolic diseases such as diabetes and cancer. In this study, treatment with AA improved symptoms of obesity‐linked metabolic syndrome, including adipocyte differentiation and elevated levels of lipids, glucose, and insulin (Figures 1, 2, 3, 4), similar to results from previous studies.

It is important to control lipid levels to manage metabolic diseases, as high lipid levels in the body cause chronic diseases. Because lipids are susceptible to oxidation, high levels of free fatty acids in the plasma and large amounts of fat stored in adipose tissues can induce oxidative stress. Inflammation from this stress can then lead to obesity (Manna & Jain, 2015). Adipose tissue dysfunction caused by hypertrophied adipocytes stimulates the secretion of proinflammatory adipokines such as leptin, resistin, visfatin, and tumor necrosis factor (TNF)‐α. In turn, the overproduction of adipokines promotes obesity‐associated metabolic diseases, including diabetes and cardiovascular disease (Katsareli & Dedoussis, 2014). Adipokines are associated with inflammation that is linked to metabolic diseases, although the specific mechanisms remain unknown. Nevertheless, PPAR‐γ and FAS have been identified as biomarkers for evaluating adipocyte differentiation and are associated with adipokine productions. When levels of PPAR‐γ, a lipid‐activated transcription factor, increase in adipose tissue, fatty acids bind to receptors and are converted to triglycerides (Evans, Barish, & Wang, 2004; Kersten, Desvergne, & Wahli, 2000). This process activates a target gene that induces the conversion of preadipocytes to mature adipocytes (Evans et al., 2004; Kersten et al., 2000). FAS is a key lipogenic enzyme that catalyzes the synthesis of long‐chain fatty acids, that is, converting acetyl‐CoA and malonyl‐CoA to palmitate, which are then stored in adipose tissue (Griffin & Sul, 2004; Wakil, Stoops, & Joshi, 1983). Results from Western blot analysis in this study revealed that AA inhibited lipid accumulation and decreased PPAR‐γ and FAS expression levels, which were initially increased during MDI‐induced adipocyte differentiation (Figure 1d). Therefore, AA may inhibit fat accumulation by suppressing the production of PPAR‐γ and FAS‐mediated cytokines. The antioxidant properties of AA may also help prevent adipocyte‐induced oxidative stress and adipokine production.

Lipid metabolic pathways, such as lipogenesis, are closely linked with insulin resistance and NAFLD. Lipid accumulation in the liver leads to impaired insulin signaling and insulin resistance, which are key symptoms of NAFLD progression (Marchesini et al., 1999; Samuel & Shulman, 2012). In our in vivo model, mice fed a HFS diet had higher levels of liver fat accumulation, fat tissue weight, TG, TC, LDL‐C, fasting glucose, serum insulin, and HOMA‐IR (Figures 2, 3, 4). However, AA treatment significantly decreased levels of liver fat, LDL‐C, serum insulin, and HOMA‐IR in these mice. These findings indicate that AA can inhibit lipid accumulation in the liver and improve insulin function in vivo, but further studies are needed to elucidate how AA affects glucose uptake or insulin signaling mechanistically. Recently, it has been reported that antioxidant component including anthocyanin regulates insulin resistance by activating the adenosine monophosphate‐activated protein kinase (AMPK) enzymes and glucose transporter 4 (GLUT‐4) translocations, and decreasing the serine phosphorylation of insulin receptor substrate 1 (IRS‐1) has been reported (Belwal, Nabavi, Nabavi, & Habtemariam, 2017). Since AA has antioxidant ability like anthocyanin, it may alleviate insulin resistance by regulating enzymes related to glucose uptake, glucose transporter, or insulin receptors. Furthermore, whereas AA inhibited adipocyte differentiation in cell culture, HFS‐treated mice that consumed AA did not decrease body weight, fat tissue weight, and TG level, although their liver fat content and liver weights decreased. Several studies have reported that oxidized tea polyphenols exert an antiobesity effect in vitro and in vivo. However, recent evidence also reported that oxidized tea polyphenols did not alleviate hyperlipidemia in rats (Wang et al., 2017). Therefore, our and previous studies have some limitations: (a) One or two concentrations were administrated; (b) the mechanisms are still unclear; and (c) It is unclear that why there is no change on fat tissue weight and blood TG level. Further studies are needed to explore the precise mechanisms of action of AA and limitations.

Epigenetic modifications, such as histone acetylation, may also regulate metabolic dysfunction. Several natural compounds found in edible plants have been reported to control epigenetic expression. For example, EGCG from green tea and curcumin from turmeric have been shown to control the acetylation of histones and nonhistone proteins, and the activity of histone acetyltransferases (HATs) and histone deacetylases (Vahid, Zand, Nosrat‐Mirshekarlou, Najafi, & Hekmatdoost, 2015). In cell culture studies, EGCG and curcumin also suppressed the activation of NF‐κB, which is associated with inflammation and the inhibition of p300/CBP expression (Choi et al., 2009; Liu, Chen, Cui, & Zhou, 2005; Shishodia, Amin, Lai, & Aggarwal, 2005). EGCG is reported to inhibit the differentiation of preadipocytes as well (Lin, Della‐Fera, & Baile, 2005). In general, the dietary consumption of bioactive compounds may affect the epigenome, activating metabolic pathways that prevent obesity and diabetes caused by lipogenesis, inflammation, and glucose metabolism.

As with EGCG and curcumin, AA can function as an epigenetic regulator. In adipose cells, AA reduced the expression of TG, SREBP‐1c, and ACC, and decreased rates of histone H3 acetylation (M. K. Kim, Kim, Kim, Lee, & Chung, 2018). AA also decreased TG, SREBP‐1c, and ACC expression in p300‐knockdown cells, indicating that p300 inhibits HAT activity and that AA inhibits lipogenesis (M. K. Kim et al., 2018). Similarly, we observed that AA inhibited HAT activity in vitro (unpublished data).

The antioxidant function of AA is important for preventing metabolic disorders. AA and its derivatives contain salicylic acid, a type of phenolic acid that has therapeutic properties such as anticancer, anti‐inflammatory, and antiobesity properties. The inhibition of NF‐κB kinase and HAT activities by AA has been demonstrated in several studies (30) (Hemshekhar, Sebastin Santhosh, Kemparaju, & Girish, 2012). Therefore, the strong antioxidant properties of AA may inhibit the markers and cytokines associated with metabolic diseases, thus mitigating fatty liver disease and insulin resistance. AA may also act as a HAT inhibitor to control the expression of lipid‐related genes via epigenetic regulation.

## 5 CONCLUSION

AA can help alleviate symptoms arising from fatty liver and insulin resistance by inhibiting lipid accumulation in cells, as well as lowering liver fat content and enhancing insulin activity in mice. Therefore, our data suggest that the AA may help to protect against the development of fatty liver and diabetes.

## CONFLICTS OF INTEREST

The authors declare no potential conflicts of interest in this study.

## ETHICAL STATEMENTS

All animal experiments were approved by the Institutional Care and Use Committee at Wonkwang University (Iksan, Republic of Korea).
